# A Single, Acute Astragaloside IV Therapy Protects Cardiomyocyte Through Attenuating Superoxide Anion-Mediated Accumulation of Autophagosomes in Myocardial Ischemia-Reperfusion Injury

**DOI:** 10.3389/fphar.2021.642925

**Published:** 2021-07-19

**Authors:** Kai-yu Huang, Yong-wei Yu, Shuai Liu, Ying-ying Zhou, Jin-sheng Wang, Yang-pei Peng, Kang-ting Ji, Yang-jing Xue

**Affiliations:** ^1^ Department of Cardiology, The Second Affiliated Hospital and Yuying Children's Hospital of Wenzhou Medical University, Wenzhou Medical University, Wenzhou, China; ^2^ Department of Endocrinology, The Second Affiliated Hospital and Yuying Children's Hospital of Wenzhou Medical University, Wenzhou Medical University, Wenzhou, China; ^3^ Department of Nephrology, The Second Affiliated Hospital and Yuying Children's Hospital of Wenzhou Medical University, Wenzhou Medical University, Wenzhou, China

**Keywords:** astragaloside IV, myocardial ischemia-reperfusion, superoxide, autophagy, apoptosis

## Abstract

Myocardial ischemia-reperfusion (I/R) injury, characterized by myocardial cell death (e.g., apoptosis) and generation of reactive oxygen species (ROS) such as superoxide (O_2_^·−^) and hydrogen peroxide (H_2_O_2_), is a serious threat to human health and property. Saponin astragaloside IV (ASIV), extracted from Chinese herbal medicine astragalus, is effective in resolving multiple pathological issues including myocardial I/R injury. Recent studies have shown that autophagy is regulated by ROS and plays an important role in myocardial I/R injury. However, regulation of autophagy by ASIV during myocardial I/R injury and the role of specific ROS involved in the process have been rarely reported. In the present study, we found that SOD2 was downregulated and O_2_^·−^ was upregulated in H_2_O_2_-induced H9C2 cardiac myocyte injury *in vitro* and myocardial I/R injury *in vivo*, while such alterations were reversed by ASIV. ASIV possessed the ability to alleviate myocardial I/R injury via attenuating I/R-caused autophagosome accumulation. Upregulate of O_2_^·−^ by 2-methoxyestradiol (2-ME) reversed the effect of ASIV-mediated autophagy regulation, which suggested that O_2_^·−^ was vital in this process. In conclusion, our results contribute to understanding the mechanism of ASIV-induced cardioprotective effect.

## Introduction

Myocardial infarction (MI) is one of the leading causes of death in the world threatening human life. Percutaneous coronary intervention (PCI) is the most effective way to treat MI (Hausenloy and Yellon, 2008). However, restoration of coronary blood flow can cause secondary injury of ischemic myocardium, termed as myocardial I/R injury (Braunwald and Kloner, 1985). Therefore, searching for strategies to alleviate myocardial I/R injury is of urgency for the treatment of MI.

Increasing studies have confirmed that autophagy is a ubiquitous pathophysiological process in myocardial I/R injury (Gustafsson and Gottlieb, 2008; Sadoshima, 2008). Autophagy is a dynamic process that degrades organelles or proteins through a lysosome-dependent pathways, and thus it has an important impact on cell survival and death (Sanchez et al., 1998). Although studies have found that autophagy induced by ischemia is beneficial while autophagy during reperfusion is destructive (Matsui et al., 2007), the role of autophagy in myocardial I/R injury is still not conclusive. For example, enhancing autophagy has been reported to both promote and inhibit cell death in myocardial I/R injury (Ma et al., 2012; Zhao et al., 2013). However, it is clear that manipulation of autophagy might be an effective way to reverse cell death during myocardial I/R injury.

A previous study has reported a maximum generation of reactive oxygen species (ROS) during the first minute of reperfusion (Ambrosio et al., 1991). Cellular ROS, including superoxide radical O_2_^•-^, hydrogen peroxide H_2_O_2_, and hydroxyl radical OH^•^, is of vital importance in the pathological process of myocardial I/R injury (De Lazzari et al., 2018). ROS is involved in multiple crucial pathways such as apoptosis, inflammation, necroptosis, and autophagy (Becker, 2004).

Saponin astragaloside IV (ASIV) is the main active ingredient extracted from *Astragalus membranaceus*. As a traditional Chinese herbal medicine, it is widely used in the treatment of various diseases in China (Xu et al., 2007). ASIV has multiple pharmacological properties, especially anti-inflammatory and anti-oxidation effects (Wang et al., 2018). In addition, ASIV can also combat myocardial I/R injury through regulation of energy metabolism and reduction of calcium load (Xu et al., 2008; Tu et al., 2013). Considering the urgency of myocardial ischemia treatment, it seems to be more necessary to investigate if a single, acute treatment of ASIV has cardioprotective effect against myocardial I/R injury. Furthermore, ASIV has been proved to mitigate various diseases through regulation of autophagy, while such an effect is still not studied in myocardial I/R injury (Guo et al., 2017; Li et al., 2018). The aim of this study is to further investigate the mechanism of ASIV against myocardial I/R injury, based on its potential regulation of autophagy.

## Materials and Methods

### Chemicals and Reagents

ASIV, triphenyltetrazolium chloride (TTC), Evans Blue, dimethyl sulfoxide (DMSO) , Chloroquine (CQ), propidium iodide (PI), Hoechst, 2-ME were obtained from Sigma-Aldrich (St. Louis, MO, United States). RIPA lysis Buffer, Bicinchoninic acid (BCA) kit and Dihydroethidium (DHE) were purchased from Beyotime (Shanghai, China). DMEM and FBS were purchased from Gibco Laboratories (Life Technologies, Inc., Burlington, ON, Canada). CCK-8 was purchased from Dojindo Laboratories (Tokyo, Japan).The commercial assay kits for the CK-MB were from Jiancheng Bioengineering (Nanjing, Jiangsu, China). The primary antibodies used were anti-glyceraldehyde-3-phosphate dehydrogenase (GAPDH) Bioworld (MN, United States), anti-Bax, anti-P62 were purchased from Cell Signaling Technology (Beverly, MA, United States). In addition, anti-LC3B from Sigma and anti-Bcl-2, anti-SOD2 from Absin were used.

### Animals and H9C2 Myocyte Culture

All animal care and experimental procedures were recognized by the Animal Care and Use Committee of Wenzhou Medical University (code number wydw 2018-0056). Male C57BL/6 mice (20–25 g), 7–8 weeks old, were obtained from the SLAC Laboratory Animal Centre of Shanghai. The mice were fed according to the breeding regulations and adapted to the environment for 1 week. H9C2 cells was obtained from the American Type Culture Collection (Manassas, VA, United States), were cultured in Dulbecco’s Modified Eagle’s Medium (DMEM) containing 4.5 g/L glucose, 10% fetal bovine serum(FBS), and 1% penicillin/streptomycin at 37°C in a humidified atmosphere.

### Myocardial I/R Protocol

Surgical procedure was performed as previously described (Wu et al., 2017). Briefly, The mice were anesthetized using 2% isofluorane. A retractor was use to open the thoracic cavity, followed by ligation of the left anterior descending (LAD) branch of the coronary artery to induce myocardial ischemia. After 30 min of ischemia, the slipknot was released for 4 h. In the ASIV groups, drug was injected intraperitoneal (i.p.) at 20 mg/kg during reperfusion (Li et al., 2012; Zhang et al., 2019). CQ (a lysosomal acidification inhibito,10 mg/kg) was administered via an intraperitoneal injection 1 h before surgery. 2-ME (a SOD inhibitor, 20 mg/kg) was dissolved in 0.5% DMSO and intraperitoneally injected after surgery (Chen et al., 2009; Schaible et al., 2014). Sham group mice experienced the same protocol without LAD occlusion. After the surgery, the mice were immediately received 10 mg/ml of pentobarbital sodium (0.1 ml/20 g) to ensure minimal pain and moved to a quiet room under gentle lighting. At the end of reperfusion, mice were euthanized after isoflurane anesthesia.

### Cell Treatment

Experiments were performed when cell density reached 80–90% confluence. Briefly, H9C2 cells were cultured with 250 µmol/L tert-butyl hydroperoxide (TBHP) for 4 h to mimic damage induced by I/R. To evaluate the protective effects of ASIV and the role of autophagy, cells were pretreated with ASIV (50 μM), CQ (10 μM) for 12 h or 2-ME(10 μM) for 24 h (Patterson et al., 2012). The exact group size for each experimental group *in vitro* is 6.

### Cell Viability Assay

CCK8 assay was used to detect cell viability. In brief, The cells were seeded into 96-well culture plates. Subsequent to treatment, the viability of cells was determined using CCK-8 kit according to the manufacturer’s instructions. The amount of cell viability were normalized to the control group which was considered as 100%.

### PI Staining and Immunofluorescence

The cardiomyocytes apoptosis rates were assayed by Hoechst 33342 and PI double staining according to the instruction manual. Mice were injected with 10 mg/kg PI prior to being sacrificed. Frozen sections were stained with Hoechst. After the treatment, dead H9C2 cells were labeled with PI. For immunofluorescence staining (LC3B, 1/250 dilution), it was performed on OCT-embedded frozen myocardial tissue sections or cell climbing slice. Fluorescence images were captured using fluorescent microscope (Olympus).Five fields were randomly selected from each sample and used to calculate the cell death rate and the expression level of LC3B.

### Measurement of CK-MB Release

The level of CK-MB were measured using respective CK-MB kits (H197; Nanjing Jiancheng, Nanjing, China), according to the manufacturer’s instructions with a microplate reader (Thermo, China).

### DHE Staining for O2·^−^ I*n Situ*

DHE was used to measure O2·− levels (Xu Y. et al., 2020). Briefly, DHE fluorescent staining on 10 μm myocardial frozen sections at 37°C for 30 min in the dark. The results were documented using a fluorescence microscope.

### Detection of Mitochondrial Superoxide I*n Vitro*

Mitochondrial superoxide *in vitro* was quantified by MitoSOX Red staining (Yeasen Biotech). In short, At the end of incubation with 5 μM MitoSOX Red reagent at 37°C for 10 min, the fluorescent intensity was detected by fluorescence microscope.

### Detecting of myocardium infarct size

TTC staining was carried out to measure the myocardial infarct size. In brief, mice were anesthetized again after 4 h of reperfusion, and the LAD was ligated again in the same location. 2% evans blue was injected into the inferior vena cava. The heart was removed quickly. OCT-embedded hearts were cut in 2 mm-thick sections and incubated for 20 min at 37°C with 1% TTC. There are three colors in the sections, including blue representing the viable non-ischemic area, red expressing the myocardium area at risk (AAR) and white presenting infarct area (INF). A percent of infarcted area over total area at risk was calculated by Image J software.

### Transmission Electron Microscopy

After establishing I/R model, myocardium (1 mm^3^ ) were incubated in 2.5% glutaraldehyde for 4 h. Following fixation in 1% osmium tetroxide for 1 h, samples were dehydrated with various concentration of alcohol. Finally, samples were embedded and stained. Transmission electron microscopy (FEI, Hillsboro, OR) was used to analyze the samples.

### Immunohistochemistry

At the end of reperfusion, hearts were excised and flushed the hearts with 0.9% saline followed by 4% paraformaldehyde. The paraffin-embedded hearts were cut into 5 μm sections and processed for immunohistochemical analysis(anti-LC3 (1:250)). The method was as described previously (Wang et al., 2018).

### Protein Preparation and Western Blotting

Western blotting was carried out in previous studies (Li et al., 2015a). Tissue or cell lysates were homogenized in RIPA buffer. Protein concentration was determined by BCA. 20–40 mg of protein was loaded for SDS-PAGE before transfered to PVDF membrane. The membranes were blocked by 5% skim milk containing 0.1% Twain 20 for 2 h, then washed. After that, the membranes were incubated with primary antibody overnight at 4°C, then washed and incubated with goat anti-rabbit IgG, peroxidase conjugated (1:10000; Biosharp, China) for 2 h. The target protein signal was detected and digitalized using ECL by using the ChemiDicTM XRS + Imaging System (Bio-Rad). Signal intensity of the protein was detected using Image J. To determine relative changes in protein expression, signal intensity of the protein was normalized to the control group or sham group which was considered as 1.

### Statistical Analysis

Statistical analysis was performed by the SPSS software 21.0. Statistically significant differences were evaluated by Student t test(for two groups) and one-way ANOVA with Tukey’s post hoc test or nonparametric Kruskal–Wallis test followed by the Bonferroni test(for multi-group). The data are expressed as mean ± SD. *p* < 0.05 was considered significant.

## Results

### ASIV Attenuates I/R-Induced Myocardial Injury

**FIGURE 1:**
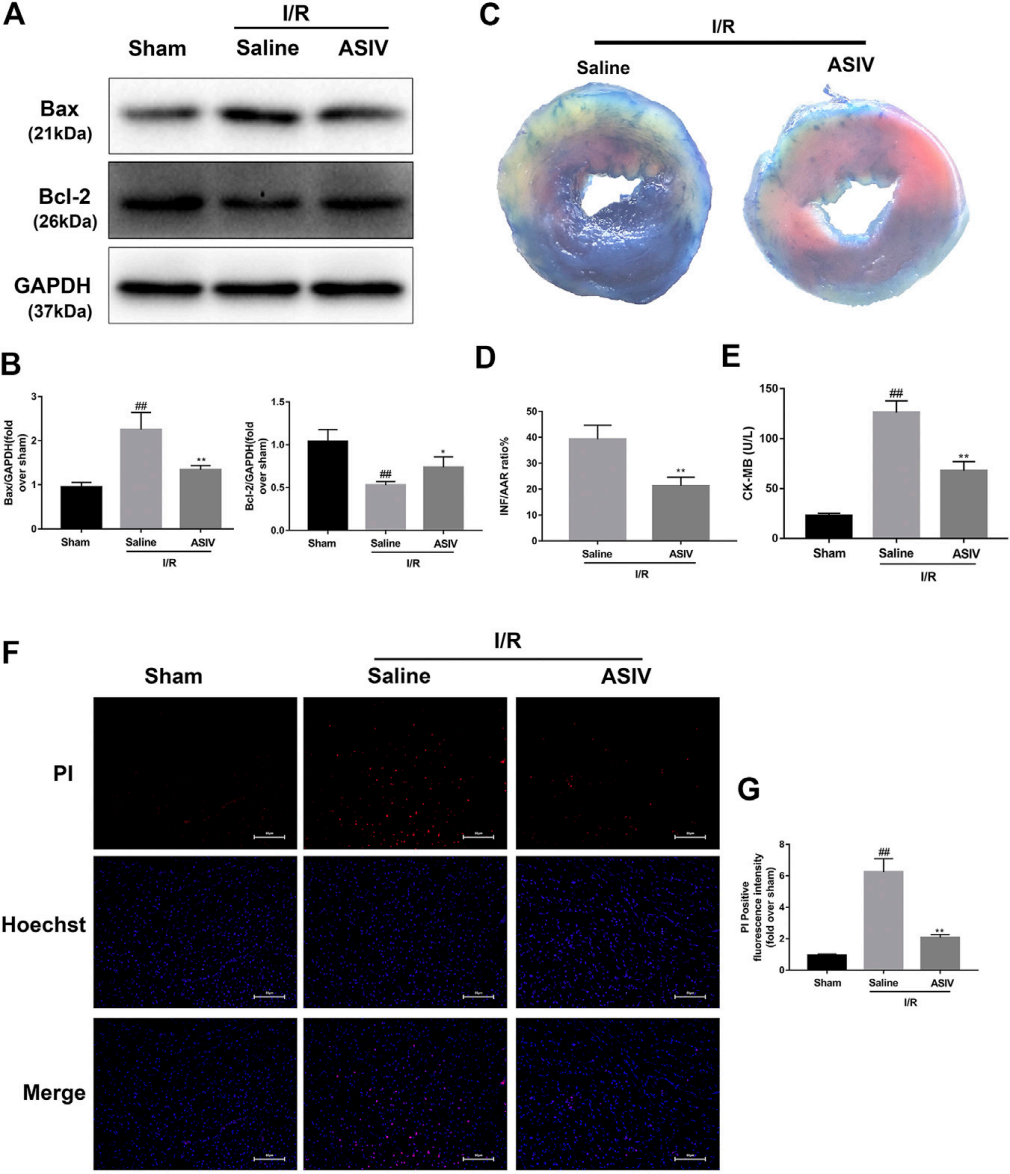
ASIV attenuates I/R injury *in vivo*. Mice were treated with ASIV (20 mg/kg; i.p.) during reperfusion, **(A, B)** western blot results of Bax and Bcl-2 in the sham group, I/R group and ASIV treatment group were analyzed, (**C, D)** TTC stain, **(E)** CK-MB release were analyzed after reperfusion for 4 h. **(F, G)** PI stain were analyzed after reperfusion for 24 h. Scale bar: 50 μm *n* = 6. Values are expressed as the means ± SD. #*p* < 0.05 compared with the sham group, *p < 0.05 compared with the I/R group (each test was repeated three times).

**FIGURE 2:**
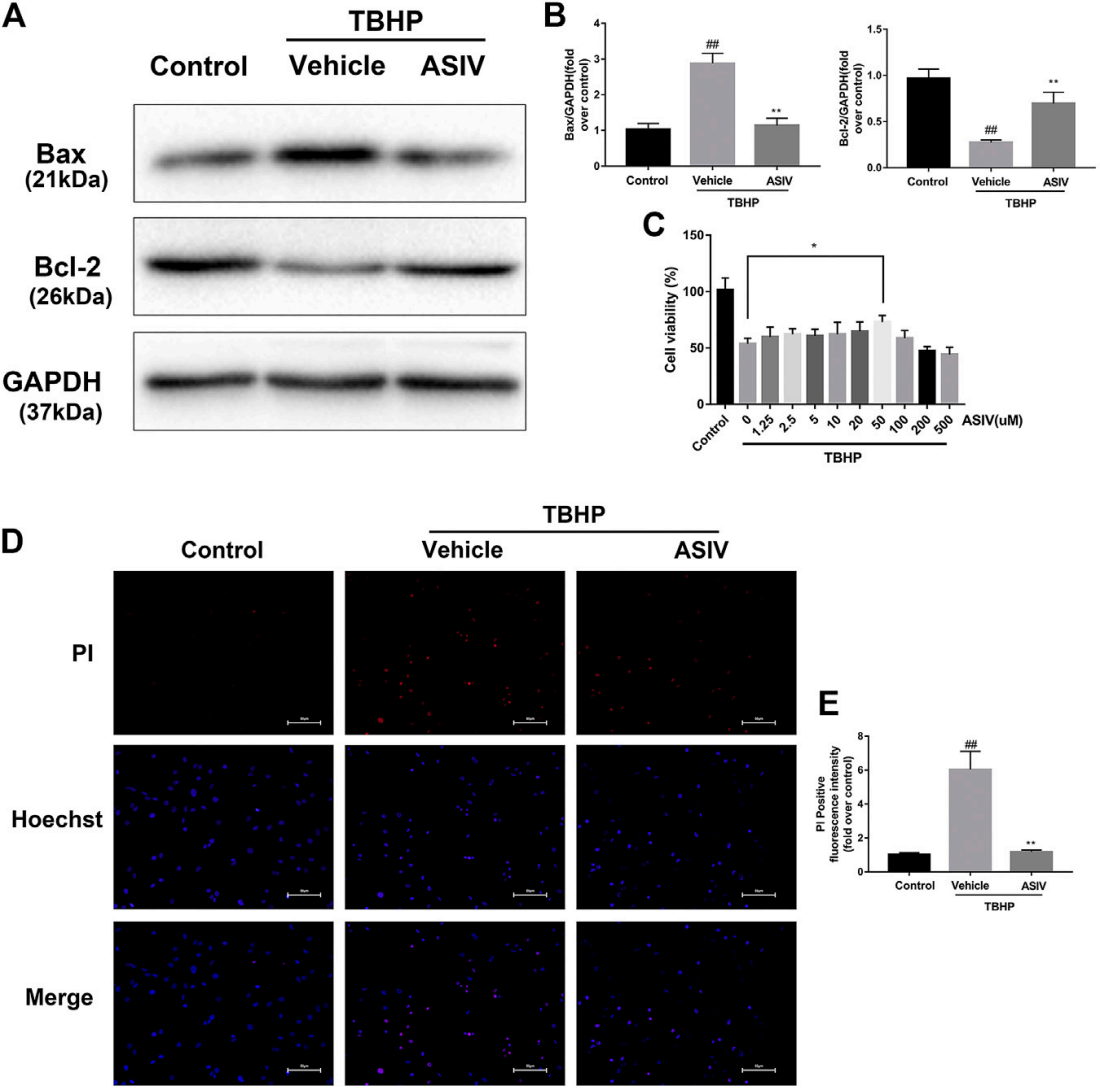
ASIV attenuates TBHP-induced H9C2 cells injury. **(A, B)** After TBHP-induced injury, western blot results of Bax and Bcl-2 in the control group, TBHP group and ASIV treatment group were analyzed. **(C)** H9C2 cells were pretreated with ASIV(1.25, 2.5, 5, 10, 20, 50, 100, 200,or 500 μM) for 12 h and then exposed to TBHP(250 μM) for 4 h, and cell viability was measured. **(D, E)** PI stain were analyzed after treated with TBHP for 4 h. Scale bar: 50 μm *n* = 6. Values are expressed as the means ± SD. #*p* < 0.05 compared with the control group, *p < 0.05 compared with the TBHP group (each test was repeated three times).

To test the efficacy of ASIV in ameliorating the response to myocardial I/R injury, mice were underwent myocardial ischemia for 30  min, followed by 4 h reperfusion. The myocardial infarct size, serum marker enzymes, and cell death level of injured myocardium were detected after reperfusion. Our findings indicate that the infarct size in mice treated with ASIV was decreased, in comparison with that in the I/R group (Figures 1C,D). As an indicator, the level of CK-MB can reflect myocardial injury. Relative to the sham group, the serum level of CK-MB was markedly elevated in the I/R group; however, this tendency was counteracted by ASIV (Figure 1E). Cardiomyocyte death is an important contributor to infarct size expansion and cardiac dysfunction following I/R injury. In order to evaluate apoptosis-related alterations, we examined the protein expression of Bax and Bcl-2. PI and Hochest 33342 double staining was used to distinguish between live and dead cells. ASIV treatment induced an increased Bcl-2 expression and decreased Bax expression (Figures 1A,B); meanwhile, the percentage of PI-positive cells was also declined (Figures 1F,G). To further evaluate the cardiac protection of ASIV, H9C2 cells were treated with TBHP *in vitro* (Figure 2). ASIV, at a dose of 50 μM, significantly increased the cell viability (Figure 2C). Furthermore, similar to the *in vivo* results, ASIV reduced the level of Bax and the ratio of PI-positive cells.

### ASIV Attenuated I/R-Induced Autophagosome Accumulation

**FIGURE 3:**
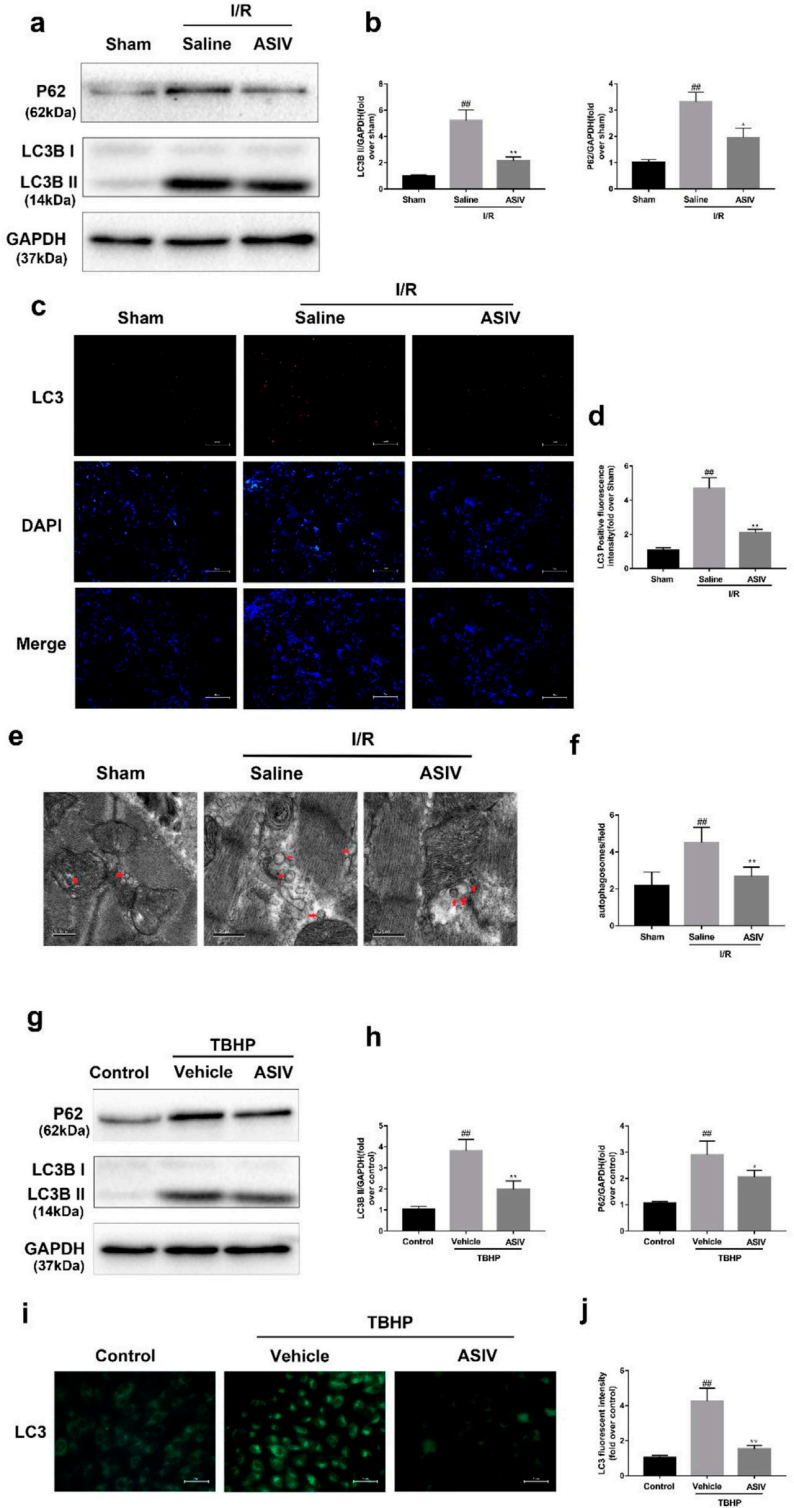
ASIV attenuated I/R-induced autophagosome accumulation. In I/R induced mice hearts after various treatment, **(A, B)** protein expression levels of LC3B and P62 were analyzed by western blot. **(C, D)** Autophagosomes (red) in cells in the Sham, I/R and ASIV groups by Immunofluorescence staining for LC3. Scale bar:100 µm. After H9C2 cells were treated with ASIV and suffered from TBHP injury, **(E, F)** Transmission electron micrographs of LV tissue sections. Transmission electron micrographs (×17,000) of the myocardium showing autophagosomes surrounded by double membranes (arrows). **(G, H)** protein expression levels of LC3B and P62 were analyzed by western blot. **(I, J)** Representative immunofluorescence images of H9C2 loaded with LC3. The autophagosomes of cells was determined. Scale bar:25 μm. *n* = 6. Values are expressed as the means ± SD. #*p* < 0.05 compared with the sham or control group, *p < 0.05 compared with the I/R or TBHP group (each test was repeated three times).

LC3 plays an essential role in autophagosome formation, and P62 is considered as a substrate in autophagic degradation, the accumulation of which suggests repressed autophagic degradation (Hou et al., 2019; Xu L. et al., 2020). As illustrated in Figure 3, the protein expression of LC3B II and P62 was increased in the I/R or TBHP-exposed group both *in vivo* and *in vitro*, which suggested that I/R injury impaired the clearance of autophagosomes. However, the expression levels of these two proteins were reduced by ASIV treatment, reinforcing the clearance of autophagosomes. To further verify this result, we examined LC3 expression in the frozen cardiac sections and in H9C2 cells through immunofluorescence analysis, and evaluated the changes in the number of autophagosomes through TEM. In consistent with the previous finding, we found that the LC3 fluorescence intensity and the number of autophagosomes rapidly increased following I/R injury and subsequently declined by ASIV treatment.

### Attenuating I/R-Induced Autophagosome Accumulation Accounts for AS-IV -Mediated Protection in Myocardial I/R Injury

**FIGURE 4:**
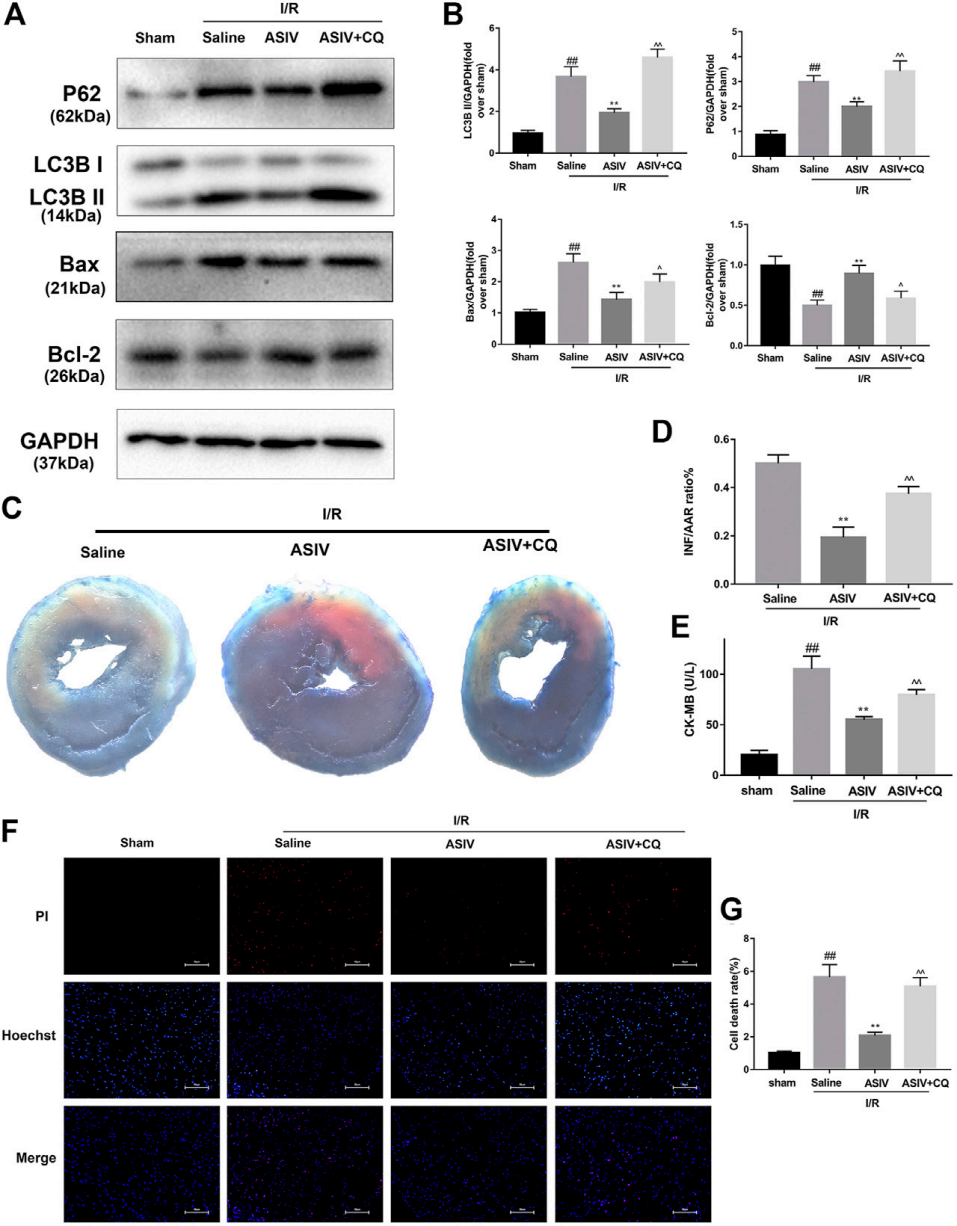
ASIV attenuated I/R injury via attenuating I/R-induced autophagosome accumulation. Mice were pretreated with CQ (10 mg/kg; i.p.)1 h before ischemia and ASIV (20 mg/kg-1; i.p.) during reperfusion, then killed at 4 h after reperfusion for detection of **(A, B)** Western blot indicating expression of LC3B,P62, Bax and Bcl-2 proteins. **(C, D)** TTC stain, **(E)** Serum CK-MB levels, **(F, G)** PI stain, scale bar: 50 μm *n* = 6. Values are expressed as the means ± SD. #*p* < 0.05 compared with the sham group, *p < 0.05 compared with the I/R group, ^*p* < 0.05 compared with the ASIV group (each test was repeated three times).

**FIGURE 5:**
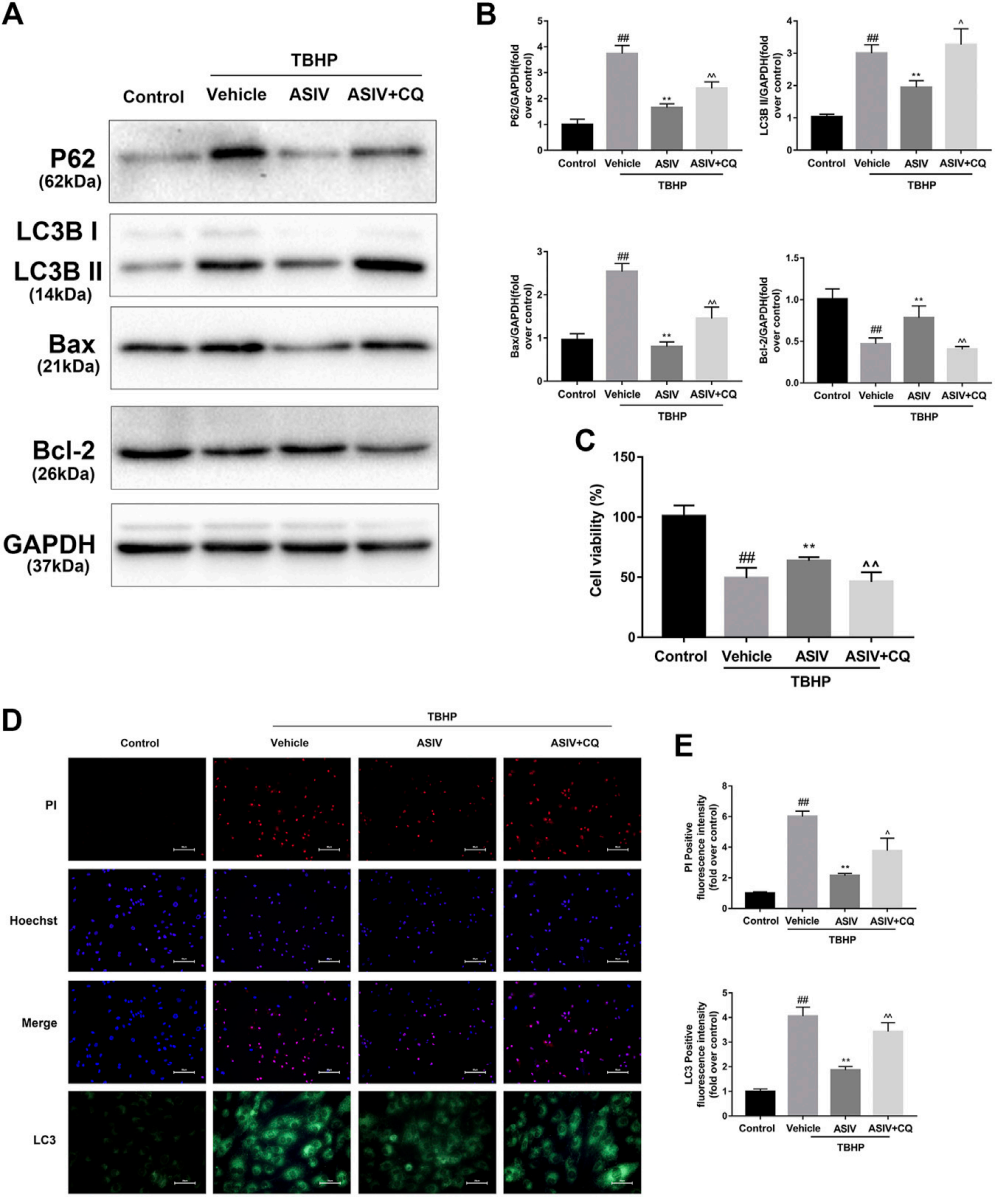
ASIV attenuated TBHP-induced injury via attenuating I/R-induced autophagosome accumulation in vitro. After H9C2 cells were pretreated with ASIV and CQ for detection of **(A, B)** Western blot indicating expression of LC3B, P62, Bax and Bcl-2 proteins. **(C)** cell viability, **(D, E)** Representative immunofluorescence images of H9C2 loaded with LC3 and PI/Hoechst stain. Scale bar: 25 μm for LC3 immunofluorescence and 50 μm for PI/Hoechst stain. *n* = 6. Values are expressed as the means ± SD. #*p* < 0.05 compared with the control group, *p < 0.05 compared with the TBHP group, ^*p* < 0.05 compared with ASIV pretreated group (each test was repeated three times).

To further examine if I/R-induced autophagosome accumulation can eventually affect cardiomyocyte survival, we examined cell death. CQ was used to block the autophagosome degradation and fusion with lysosomes (Subirada et al., 2019). Through both *in vivo* and *in vitro* investigations, we found that CQ-treated mice or H9C2 cells were associated with an increased expression level of LC3B II, P62, and Bax while a decreased expression level of Bcl-2 (Figures 4, 5). This result was further confirmed by immunofluorescence analysis of LC3 through Hochest and PI double staining, which demonstrated the potential relationship between autophagosome accumulation and cell death. Consistent with the abovementioned results, ASIV significantly reduced myocardial infarct size and promoted cell viability, which was reversed by CQ treatment. Taken together, these data suggested that attenuating the accumulation of autophagosomes during I/R injury might be the underlying mechanisms of the protective effects of ASIV.

### ASIV Decreased I/R-Caused O2•−accumulation

**FIGURE 6:**
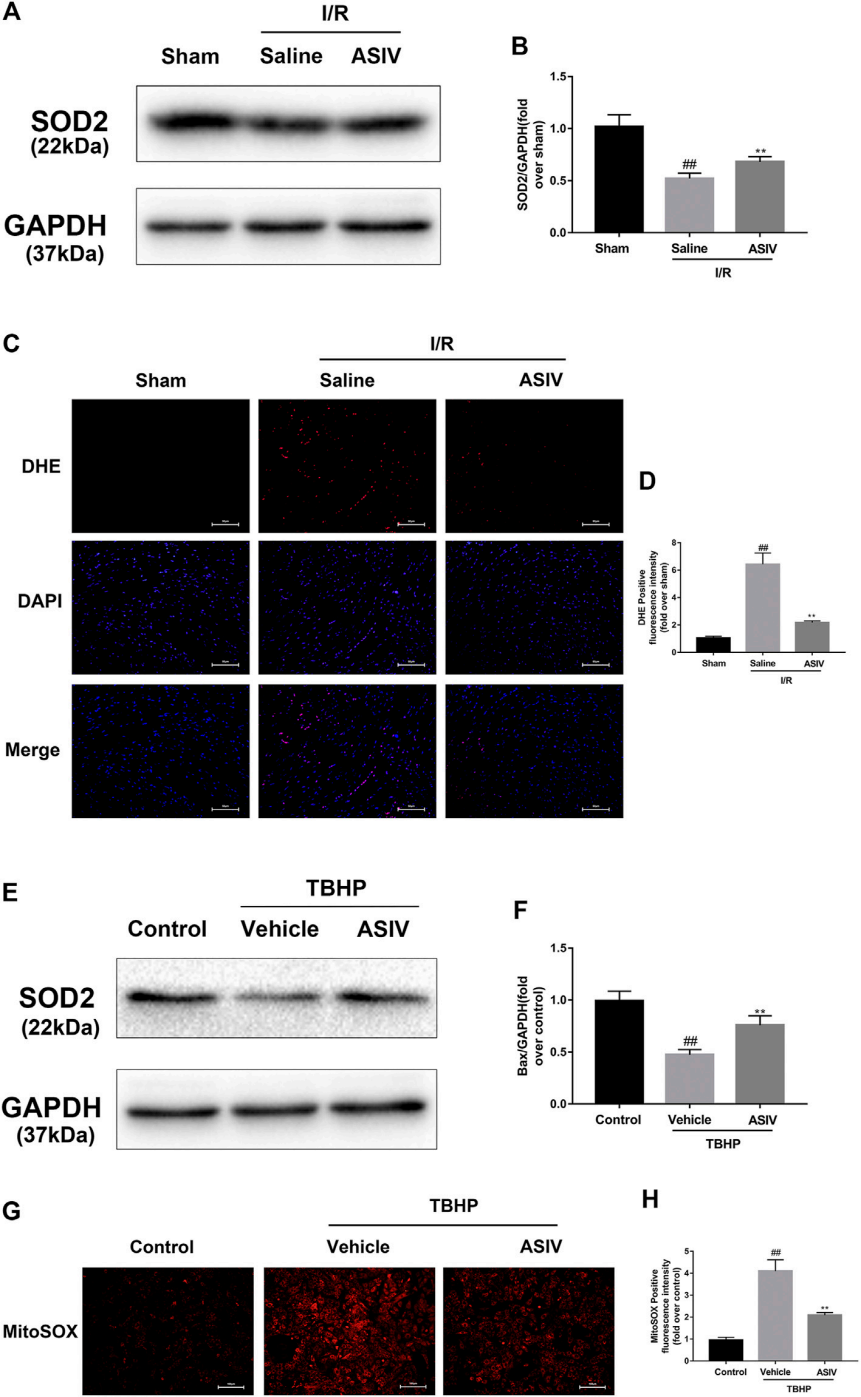
ASIV decreased I/R-caused O2•^−^ accumulation. Mice were treated with ASIV (20 mg/kg; i.p.) during reperfusion, **(A, B)** protein expression levels of SOD2 were analyzed by western blot. **(C, D)** O2•^−^ in myocardium was detected by DHE staining. Scale bar: 50 µm H9C2 cells were treated with ASIV and suffered from TBHP injury, **(E, F)** protein expression levels of SOD2 were analyzed by western blot. **(G, H)** O2•− in H9C2 cells was detected by MitoSox Red staining. Scale bar:100 µm *n* = 6. Values are expressed as the means ± SD. #*p* < 0.05 compared with the sham or control group, *p < 0.05 compared with the I/R or TBHP group (each test was repeated three times).

O_2_^·−^ is the major ROS regulator of autophagy (Chen et al., 2009). To assess the level of O_2_^·−^, we examined the protein level of SOD2 (a scavenger of O_2_^·−^) (Chen et al., 2009) through DHE (*in situ*) and MitoSOX Red (*in vitro*) staining. The generation of O_2_^•−^ was increased in mice I/R myocardium and TBHP-treated H9C2 cells (Supplementary Figure 1). As shown in Figure 6, the expression of SOD2 protein in the I/R group was markedly decreased after reperfusion, whereas ASIV remarkably promoted the activation of SOD2. Furthermore, fluorescence results also demonstrated that ASIV treatment markedly reduced the level of O_2_^•−^. These results suggested that ASIV might act as a superoxide anion scavenger during myocardial I/R injury.

### O2•− Accounts for ASIV -Mediated Regulation of Autophagy in Myocardial I/R Injury

**FIGURE 7:**
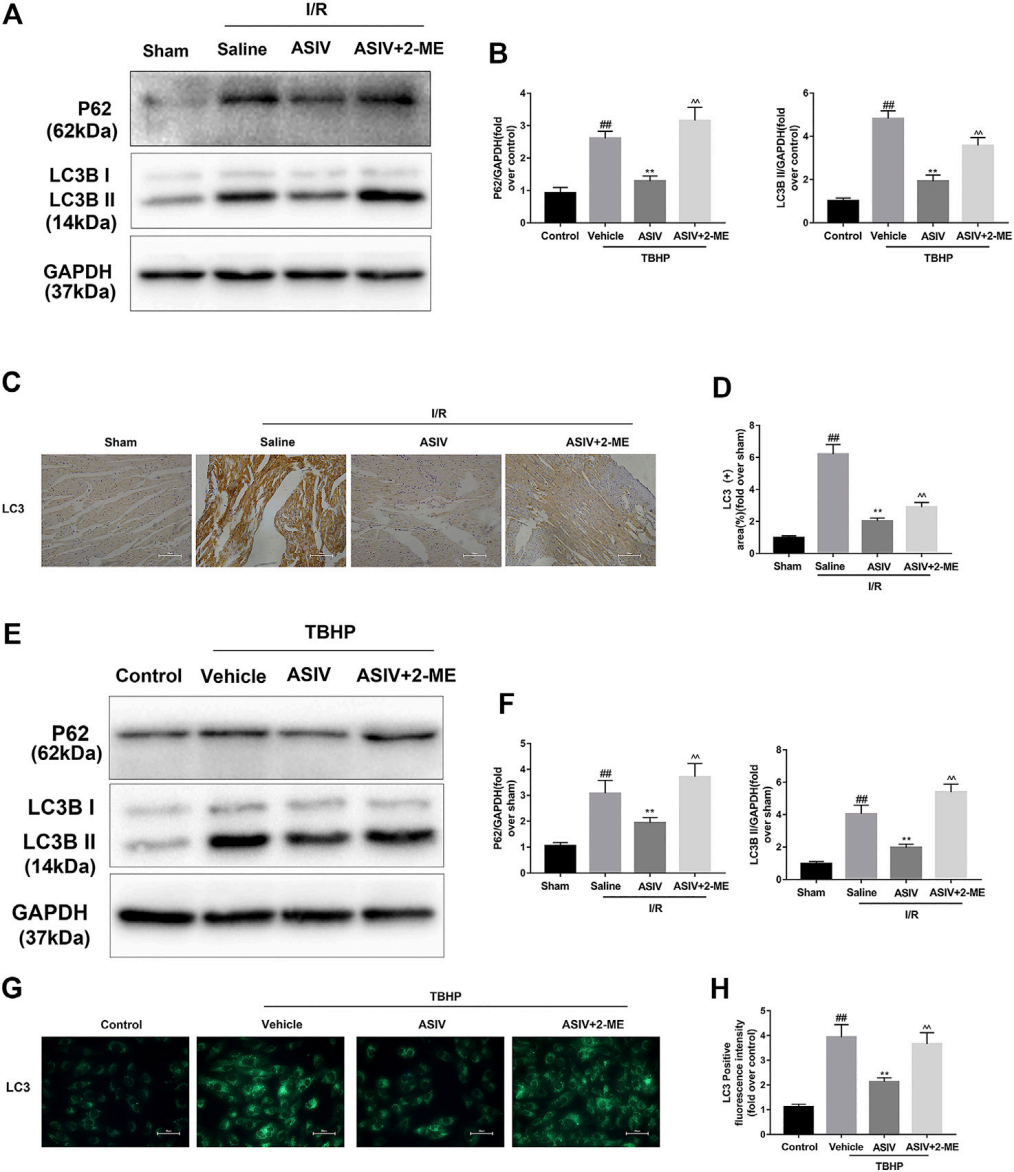
O2•− accounts for ASIV -mediated regulation of autophagy in myocardial I/R injury. Mice were treated with 2-ME (20 mg/kg; i.p.) and ASIV (20 mg/kg; i.p.) during reperfusion, then killed at 4h after reperfusion for detection of **(A, B)** Western blot indicating expression of LC3 and P62 proteins. **(C, D)** Representative IHC images of LC3B in mice myocardial cross sections (×400), **(E, F)** Western blot indicating expression of LC3B and P62 proteins, **(G, H)** Representative immunofluorescence images of H9C2 loaded with LC3, scale bar: 50 μm *n* = 6. Values are expressed as the means ± SD. #*p* < 0.05 compared with the sham or control group, *p < 0.05 compared with the I/R or TBHP group, ^*p* < 0.05 compared with the ASIV group (each test was repeated three times).

Given the essential role of autophagy in ASIV-mediated cardiac protection, we further examined if O2^•−^ plays an important role as a bridge between autophagy and ASIV, through using an O2^•−^ inducer, 2-ME (Kachadourian et al., 2001). As shown in Figure 7, the expression of LC3B II and P62 was detected by western blot. It was clear that 2-ME treatment markedly increased the level of LC3B II and P62 in both mice heart and H9C2 cells. These data suggest that ASIV reduced the accumulation of autophagosomes by down-regulatingthe level of O2^•−^. In addition, a similar result was observed from immunohistochemistry (Figures 7C,D) and immunofluorescence (Figures 7G,H) analyses.

## Discussion

**FIGURE 8:**
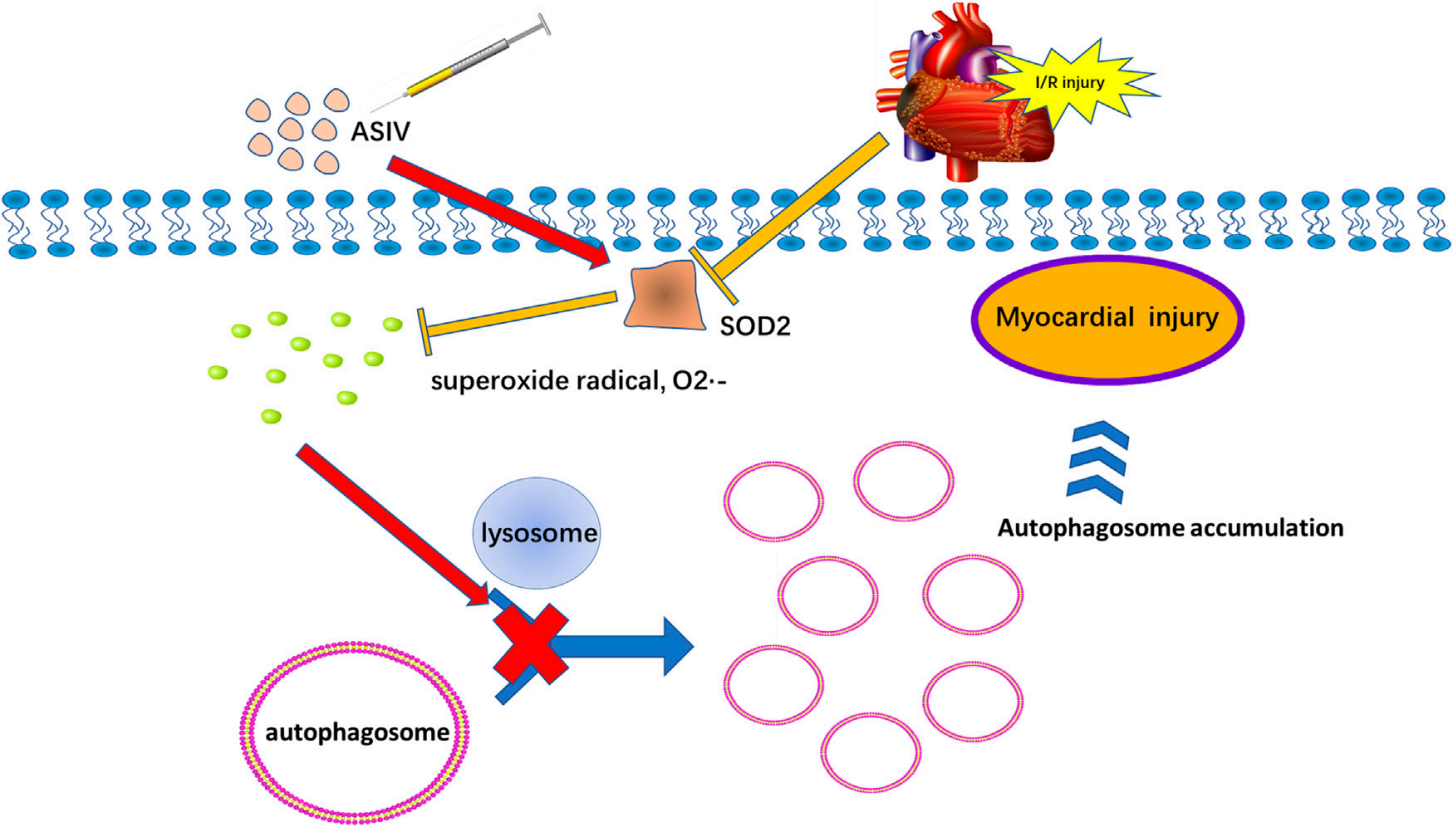
Potential mechanism of ASIV on myocardial ischemia-reperfusion injury via attenuating superoxide anion-mediated accumulation of autophagosomes.

Our results suggested that a single, acute treatment of ASIV protected against myocardial I/R injury by reducing excessive autophagy. In addition, this effect is also achieved by repressing superoxide anion (Figure 8).

MI is associated with a high mortality rate. As an effective treatment, revascularization by PCI can cause myocardial I/R injury. At the initial stages of the disease, the occurrence of oxidative stress is common, which serves as an emerging therapeutic target (Ye et al., 2020). Therefore, early intervention is proposed to alleviate the extent of ROS burst. In this study, the administration of ASIV was a single, acute therapy during reperfusion, which allowed an early intervention for the disease.

Multiple signaling pathways were involved in ASIV’s autophagy regulation, including ERK, mTOR, and Nrf2 pathways (Jiang et al., 2019; Song et al., 2019; Zheng et al., 2020). Manipulating Nrf2 pathway is considered as a beneficial strategy for inhibiting oxidative stress and cell survival (Cadenas, 2018). In the non-classical pathway, several mechanisms (e.g., regulating autophagic receptor p62) can isolate Keap1 from Nrf2 and induce downstream antioxidant effect (Kageyama et al., 2018). The suppressed autophagy leads to increased p62 accumulation, p62-Keap1 interaction, and Nrf2 nuclear translocation (Zheng et al., 2020). Activation of the Akt/mTOR pathway is crucial for the regulation of autophagy. In H9C2 cells, Zhang et al. have reported that Urolithin B can activate Akt/mTOR pathway and inhibit I/R-induced autophagy via p62/Keap1/Nrf2 signaling axis (Zheng et al., 2020). Furthermore, previous studies have confirmed that autophagy is specifically mediated by O_2_^•−^, but not by H_2_O_2_ (Chen et al., 2009; Pi et al., 2015). Oxidative stress is involved in multiple types of cell injuries and controlled by antioxidant enzymes such as SOD. The role of SOD2 as an antioxidant enzyme has been well documented, which can transform O_2_^•−^ into H_2_O_2_ (Chen et al., 2009). In the present study, ASIV treatment significantly increased SOD2 level and decreased the accumulation of autophagosomes, thereby attenuating cell death induced by I/R, indicating that downregulation of O_2_^•−^ is an important potential mechanism for autophagy. H_2_O_2_, as a ubiquitous ROS, can be catalyzed to H_2_O by antioxidant enzymes (catalase, GPx, and PrxIII) (Chen et al., 2009). A number of studies have already shown that H_2_O_2_ could also induce autophagy (Hariharan et al., 2011; Li et al., 2015b). In our study, we treated the H9C2 cells exogenously with TBHP to mimic I/R injury *in vitro*. The results demonstrated that TBHP induced O_2_^•−^ accumulation in a time-dependent manner, which is in line with the findings from a previous study that exogenous H_2_O_2_ increased the generation of intracellular O_2_^•−^(Chen et al., 2009). We also observed that ASIV markedly reduced O_2_^•−^ level and the accumulation of autophagosomes, whereas this effect was partially reversed in the presence of 2-ME(O_2_^•−^ inducer). Considering that the system is in a chain reaction between O_2_^•−^and H_2_O_2_ (Azad et al., 2009), SOD2 inhibition might induce a significant increase of O_2_^•−^, corresponding with a significant decrease of H_2_O_2_. On the other hand, this also illustrates that O2•− might play a more critical role in autophagy regulation as compared to H2O2.

Impaired autophagosome clearance contributes to various types of diseases (Sarkar et al., 2014; Cheng et al., 2019). ROS can initiate autophagosome accumulation by functioning as cellular signaling molecules (Ma et al., 2012; Wang et al., 2019). However, the role of ROS (mainly O_2_^•−^) in ASIV-mediated regulation of autophagy remains largely unknown. As discussed previously, our results indicate that ASIV relieves the accumulation of O_2_^•−^ and autophagosomes, thus protecting cardiomyocytes from I/R injury. CQ prevents the fusion between autophagosomes and dilated lysosomes by inducing lysosomal alkalinization (Lu et al., 2016). We found that CQ significantly induced autophagosome accumulation and reversed the protective effects of ASIV, suggesting that attenuating I/R-induced autophagosome accumulation contributes to the ASIV-mediated protection in myocardial I/R injury. Mechanistically, we found that ASIV treatment reduced I/R-induced autophagosome accumulation induced by myocardial I/R injury, which might be attributed to the inhibition of O_2_^•−^. Additionally, O_2_^•−^ was proved to upregulate PI3K class III, which could further enhance the formation of autophagosomes (Chen et al., 2017). Moreover, another study demonstrated that ROS can upregulate beclin-1 and reduce LAMP2 during myocardial I/R injury, and their combined effect blocks autophagosome degradation (Ma et al., 2012). Either increased formation or suppressed degradation of autophagosomes can aggravate autophagosome accumulation. However, further exploration and verification are required to understand how ASIV downregulates O_2_^•−^.

## Conclusion

This study showed that myocardial I/R injury enhanced accumulation of O2•− and autophagosomes. A single, acute ASIV treatment would reduce myocardial I/R injury through attenuating superoxide anion-mediated accumulation of autophagosomes. At last, we believe that our results provide a completely new view on the molecular mechanisms and potential cardiac therapeutic benefits of ASIV.

## Data Availability

The raw data supporting the conclusions of this article will be made available by the authors, without undue reservation, to any qualified researcher.
