# Correction: A single, acute astragaloside IV therapy protects cardiomyocyte through attenuating superoxide anion-mediated accumulation of autophagosomes in myocardial ischemia-reperfusion injury

**DOI:** 10.3389/fphar.2026.1790478

**Published:** 2026-03-24

**Authors:** Kai-yu Huang, Yong-wei Yu, Shuai Liu, Ying-ying Zhou, Jin-sheng Wang, Yang-pei Peng, Kang-ting Ji, Yang-jing Xue

**Affiliations:** 1 Department of Cardiology, The Second Affiliated Hospital and Yuying Children's Hospital of Wenzhou Medical University, Wenzhou Medical University, Wenzhou, China; 2 Department of Endocrinology, The Second Affiliated Hospital and Yuying Children's Hospital of Wenzhou Medical University, Wenzhou Medical University, Wenzhou, China; 3 Department of Nephrology, The Second Affiliated Hospital and Yuying Children's Hospital of Wenzhou Medical University, Wenzhou Medical University, Wenzhou, China

**Keywords:** astragaloside IV, myocardial ischemia-reperfusion, superoxide, autophagy, apoptosis

There was a mistake in Figure 3 as published. The electron microscopy image for the Sham group in Figure 3E and the cell fluorescence image for the ASIV group in Figure 3I were inadvertently duplicated with images from our previous study. The corrected Figure 3 appears below.

**FIGURE 3:**
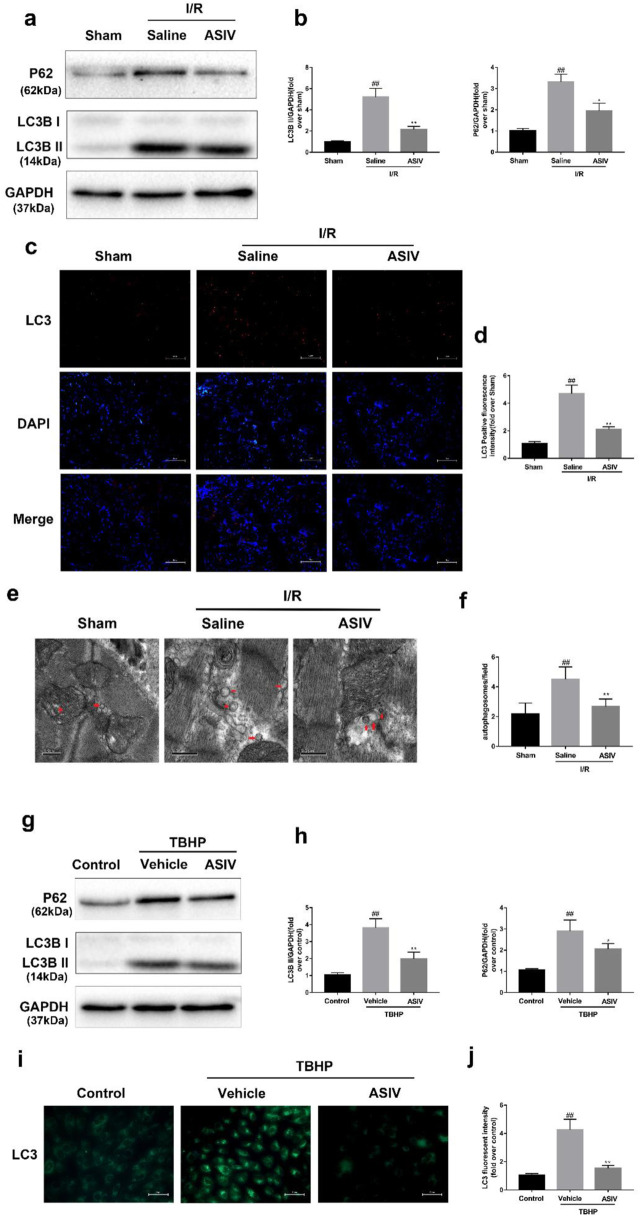
ASIV attenuated I/R-induced autophagosome accumulation. In I/R induced mice hearts after various treatment, **(A, B)** protein expression levels of LC3B and P62 were analyzed by Western blot. **(C, D)** Autophagosomes (red) in cells in the Sham, I/R and ASIV groups by Immunofluorescence staining for LC3. Scale bar: 100 µm. After H9C2 cells were treated with ASIV and suffered from TBHP injury, **(E, F)** Transmission electron micrographs of LV tissue sections. Transmission electron micrographs (×17,000) of the myocardium showing autophagosomes surrounded by double membranes (arrows). **(G, H)** protein expression levels of LC3B and P62 were analyzed by Western blot. **(I, J)** Representative immunofluorescence images of H9C2 loaded with LC3. The autophagosomes of cells was determined. Scale bar: 25 μm. *n* = 6. Values are expressed as the means ± SD. #*p* < 0.05 compared with the sham or control group, *p < 0.05 compared with the I/R or TBHP group (each test was repeated three times).

The original article has been updated.

