# Supplementary figures and images for: A Single, Acute Astragaloside IV Therapy Protects Cardiomyocyte Through Attenuating Superoxide Anion-Mediated Accumulation of Autophagosomes in Myocardial Ischemia-Reperfusion Injury
Source: Front Pharmacol. 2021 Jul 19;12:642925. doi: 10.3389/fphar.2021.642925 (PMC8327213; doi:10.3389/fphar.2021.642925)

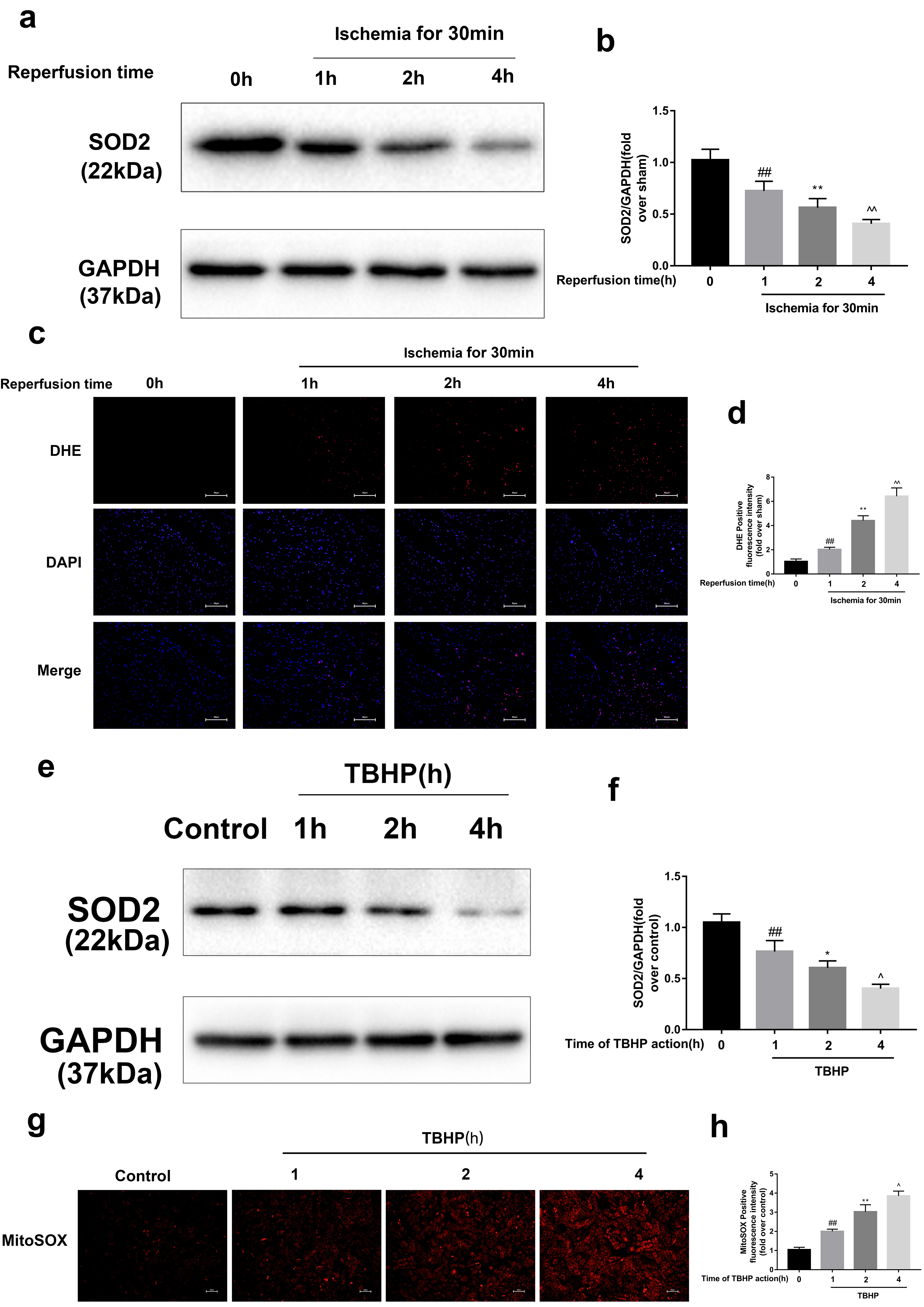

Supplement: Supplementary file 1 [file Image1.JPEG]
