# Supplementary material for: A Single, Acute Astragaloside IV Therapy Protects Cardiomyocyte Through Attenuating Superoxide Anion-Mediated Accumulation of Autophagosomes in Myocardial Ischemia-Reperfusion Injury
Source: Front Pharmacol. 2021 Jul 19;12:642925. doi: 10.3389/fphar.2021.642925 (PMC8327213; doi:10.3389/fphar.2021.642925)
Supplement: Supplementary file 2 [file DataSheet1.PDF]

Supplementary data

A single, acute Astragaloside IV therapy protects cardiomyocyte through attenuating superoxide anion-mediated accumulation of autophagosomes in myocardial ischemia-reperfusion injury

Kai-yu Huang1 $, Yong-wei Yu 1$, Shuai-Liu1, Ying-ying Zhou2, Jin-sheng Wang1, Yang-pei Peng3, Kang-ting Ji1# and Yang-jing Xue1#
1Department of Cardiology, The Second Affiliated and Yuying Children's Hospital of Wenzhou Medical University, Wenzhou, Zhejiang, 325000, China
2Department of Endocrinology, The Second Affiliated and Yuying Children's Hospital of Wenzhou Medical University, Wenzhou, Zhejiang, 325000, China
3Department of Nephrology, The Second Affiliated and Yuying Children's Hospital of Wenzhou Medical University, Wenzhou, Zhejiang, 325000, China
$These two authors contributed equally to this work.
# Co-corresponding Authors:
Kang-ting Ji, MD, Department of Cardiology, The Second Affiliated Hospital and Yuying Children's Hospital of Wenzhou Medical University, Xueyuanxi Road, No 109, Wenzhou, 325000, Zhejiang, China.Tel: 86-577-88002214; Fax: 86-577-88002214; E-mail:jikt@wmu.edu.cn
Yang-jing Xue, MD, Department of Cardiology, The Second Affiliated Hospital and Yuying Children's Hospital of Wenzhou Medical University, Xueyuanxi Road, No 109, Wenzhou, 325000, Zhejiang, China.Tel: 86-577-88002214; Fax: 86-577-88002214; E-mail: 497486104@qq.com

Supplementary Figure S1. The generation of O2•− is increased in mice I/R myocardium and TBHP treated H9C2 cells. As expected,SOD2 expression decreases with reperfusion time , following 30min ischemia or TBHP exposure time both in frozen myocardial tissue sections and H9C2 cells. Next, DHE and MitoSox Red fluorescence intensity also clearly demonstrated the time dependent changes in O2·−.

Supplementary figure S1 legends

The generation of O2•− is increased both in vivo and in vitro. Following 30 min ischemia, mice were suffered from various reperfusion time, (a, b)protein expression levels of SOD2 were analyzed by western blot. (c, d) O2•− in myocardium was detected by DHE staining. Scale bar:50μm. After H9C2 cells were Incubated with TBHP for various time, (e, f)protein expression levels of SOD2 were analyzed by western blot. (g, h) O2•− in H9C2 cells was detected by MitoSox Red staining. Scale bar:100μm. n

= 6. Values are expressed as the means ± SD. #$p < 0.05$ compared with the sham or control group, *$p < 0.05$ compared with the reperfusion for 1h or TBHP for 1h group, ^$p < 0.05$ compared with the reperfusion for 2h or TBHP for 2h group (each test was repeated three times).
